# Specific Glioma Prognostic Subtype Distinctions Based on DNA Methylation Patterns

**DOI:** 10.3389/fgene.2019.00786

**Published:** 2019-09-12

**Authors:** Xueran Chen, Chenggang Zhao, Zhiyang Zhao, Hongzhi Wang, Zhiyou Fang

**Affiliations:** ^1^Anhui Province Key Laboratory of Medical Physics and Technology; Center of Medical Physics and Technology, Hefei Institutes of Physical Science, Chinese Academy of Sciences, Hefei, China; ^2^Hefei Cancer Hospital, Chinese Academy of Sciences, Hefei, China; ^3^University of Science and Technology of China, Hefei, China

**Keywords:** glioma, consensus clustering, DNA methylation, molecular subtypes, prognosis

## Abstract

DNA methylation is an important regulator of gene expression and may provide an important basis for effective glioma diagnosis and therapy. Here, we explored specific prognosis subtypes based on DNA methylation status using 653 gliomas from The Cancer Genome Atlas (TCGA) database. Five subgroups were distinguished by consensus clustering using 11,637 cytosines preceding a guanosine (CpGs) that significantly influenced survival. The specific DNA methylation patterns were correlated with age, tumor stage, and prognosis. Additionally, weighted gene co-expression network analysis (WGCNA) analysis of CpG sites revealed that 11 of them could distinguish the samples into high- and low-methylation groups and could classify the prognostic information of samples after cluster analysis of the training set samples using the hierarchical clustering algorithm. Similar results were obtained from the test set and 12 glioma patients. Moreover, *in vitro* experiments revealed an inverse relationship between methylation level and migration ability or insensitivity to temozolomide (or radiotherapy) of glioma cells based on the final prognostic predictor. Thus, these results suggested that the model constructed in this study could provide guidance for clinicians regarding the prognosis of various epigenetic subtypes.

## Introduction

Glioma derives from glial cells and is the most prevalent primary central nervous system malignant tumor (Aldape et al., 2003; Aquilanti et al., 2018). The overall survival time continues to be unsatisfactory, especially for high-grade glioma, although treatment strategies, including surgical resection, radiation, and chemotherapy, for glioma patients have been greatly improved (Jain, 2018; Zang et al., 2018). It is therefore urgent to elucidate the molecular mechanisms underlying glioma tumorigenesis for developing novel therapies.

Epigenetics is recognized as heritable alterations in gene expression not connected to an alteration in DNA sequence but plays a crucial role in carcinogenesis (El-Osta, 2004; Issa, 2007; Hao et al., 2017). Cancer epigenetics covers aspects of aberrant DNA methylation, dysregulated non-coding RNA, and altered post-translational histone modification, among which aberrant DNA methylation is most widely investigated (Dawson and Kouzarides, 2012; Kanwal et al., 2015). Aberrant DNA methylation could influence the key genes that are involved in glioma carcinogenesis and progression and may especially influence some tumor suppressor genes by altering their expression and inhibiting their function (Liu et al., 2016; Charlet et al., 2017). Thus, biological processes, specifically alterations in DNA methylation, can provide an important basis for early diagnosis and prognosis of cancer and development of new approaches for further clinical applications. Although the effects of certain genes with aberrant DNA methylation on glioma have been reported extensively, the comprehensive profile of the interaction network still needs further elucidation.

During the last decades, bioinformatics analysis and microarray technology have been widely used to identify general genetic or epigenetic alterations in carcinogenesis and screen biomarkers for prognosis and diagnosis of cancer (Crispatzu et al., 2017; Yang et al., 2019). Several single genes whose global methylation status correlates with glioma outcome and gene expression level have already been identified (Fanelli et al., 2008; Hill et al., 2014). Additionally, some research on aberrant DNA methylation has been conducted to identify glioma DNA methylation subtypes by DNA methylation profile (Gustafsson et al., 2018; Johannessen et al., 2018); however, this classification was not detailed enough, and the specific sites that are associated with each category are unclear.

In this study, we addressed glioma classification by identifying specific prognosis subtypes based on DNA methylation profiles of glioma obtained from The Cancer Genome Atlas (TCGA) database. This classification system may help identify molecular subtypes or new glioma markers to subdivide glioma patients more accurately. Moreover, our classification system provides guidance for clinicians on personalized treatments and diagnoses by identifying differences in prognosis for each epigenetic subtype.

## Materials and Methods

### Data Pre-processing and the Initial Screening of DNA Methylation Loci in Glioma

Lower-grade glioma (LGG) and glioblastoma multiforme (GBM) DNA methylation data generated with the Illumina Infinium HumanMethylation450 BeadChip array were downloaded from the TCGA data portal (Weinstein et al., 2013). Methylation level of each probe was represented by the β-value, which ranges from 0 to 1, corresponding to unmethylated and fully methylated, respectively. Probes with missing data in more than 70% of the samples were removed. The remaining probes that were not available (NAs) were imputed using the k-nearest neighbors (knn) imputation procedure. The ComBat algorithm in sva *R* package was used to remove batch effects by incorporating patient ID information and batch and integrating all the DNA methylation array data. Unstable genomic sites, including cytosines preceding a guanosine (CpGs) in single nucleotide polymorphisms and sex chromosomes, were removed. We selected CpGs in promoter regions because DNA methylation in promoter regions influences gene expression strongly. Promoter regions were defined as 2 kb upstream to 0.5 kb downstream from transcription start sites. Finally, we selected samples having gene expression profiles. In total, 653 gliomas were used for the analysis.

Next, we separated the data set into two cohorts: a training set and a test set. The criteria for this grouping were as follows: a) random division of samples into two groups and b) similar age distribution, staging, follow-up time, and death ratio in the two groups.

### Determining Classification Features by COX Proportional Risk Regression Models

CpG sites influencing survival significantly were used as classification features. First, univariate COX proportional risk regression models were constructed with methylation levels of each CpG site, age, and stage, and survival data of the cases. Then, the significant CpGs obtained from univariate COX proportional risk regression models were introduced into multivariate COX proportional risk regression models, using tumor stage and age as covariates, which were also significant in the univariate models. Finally, the CpG sites that were still significant were used as classification features. COX proportional hazard models were fitted with methylation levels of CpGs using the coxph function in survival package *R*, with clinical and demographic attributes (stage and age) as covariates in the multivariate analysis.

### Consensus Clustering to Obtain Molecular Subtypes Associated With Glioma Prognosis

Consensus clustering was performed with the ConsensusClusterPlus package in *R* to determine subgroups of gliomas based on the most variable CpG sites (Wilkerson and Hayes, 2010). In this study, 80% of the samples were sampled 100 times by adopting the resampling program; the similarity distance between samples was estimated by the Euclidean distance (Ghosh and Barman, 2016), and kmdist was used as the clustering algorithm to search for the reliable and stable subgroup classification. After executing ConsensusClusterPlus, the item-consensus results and cluster consensus were obtained. The criteria to determine the number of clusters were as follows: relatively high consistency within clusters, relatively low variation coefficient, and no appreciable rise in the area under the cumulative distribution function (CDF) curve. Variation coefficient was calculated according to the following formula: coefficient of Variation (CV) = (SD/MN)*100%, where MN represents the average of samples and SD represents the standard deviation. The category number was selected as the area under the CDF curve and showed no significant change. The heat map corresponding to the consensus clustering was generated by pheatmap *R* package.

### Survival and Clinical Characteristic Analyses

Kaplan–Meier plots were used to determine overall survival among glioma subgroups defined by DNA methylation profiles. The log-rank test was used to measure the significant differences among the clusters. Survival analyses were performed with the survival package in *R* software. Associations between biological and clinical characteristics and DNA methylation clustering were analyzed with the chi-square test. All tests were two-sided, and for all statistical tests, *p* < 0.05 was considered to be significant unless otherwise noted.

### Glioma Cell Survival and Migration Assays

After receiving informed consent, glioma specimens were obtained from patients undergoing surgery at the Hefei Cancer Hospital, Chinese Academy of Sciences, in accordance with the Institutional Review Board. Within hours after surgical removal, tumor specimens were enzymatically dissociated into single cells, following previously reported procedures (Chen et al., 2016). For cell survival assay, the cells were plated at a seeding density of 10,000 cells/plate in a 60 mm plate, treated with or without temozolomide or 6 Gy radiotherapy, grown for 48 h in a standard growth medium, and washed with phosphate buffer saline (PBS). For cell migration assay, cell suspension in serum-free medium was added to the upper Transwell chamber and then incubated for 18 h. The cells were fixed in cold methanol for 20 min, washed, and stored. Fixed cell colonies were visualized by incubating the cells with 0.5% (w/v) crystal violet for 0.5 h. Excess crystal violet was removed by washing with PBS. Cells that survived or migrated were counted. Differences in means were considered statistically significant when *p* < 0.05 using a two-tailed *t* test.

## Results

### DNA Methylation Features for Classification Based on Prognosis

**Figure 1:**
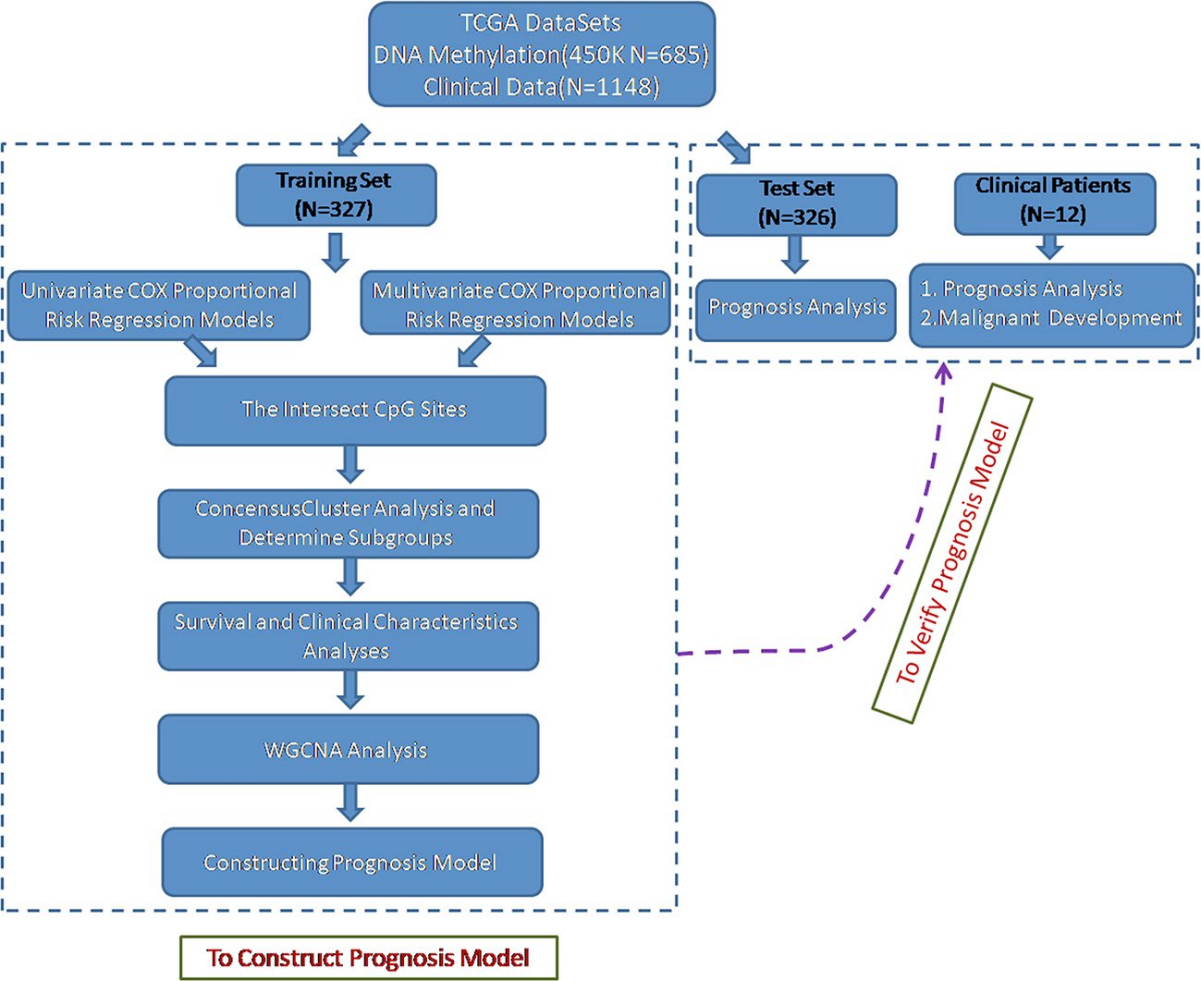
Flowchart describing the schematic overview of the study design.

To identify the specific CpG sites that were significantly correlated with survival in glioma, we set up the workflow shown in **Figure 1**. The 450 k methylation profiles were downloaded from *TCGA*; 485,577 CpG sites in 685 samples and clinical follow-up information from 1,148 cases were obtained. There were 653 matched samples between clinical data and methylation profiles. The samples were evenly divided into a training set (n = 327) and test set (n = 326); four properties (including age, follow-up period, proportion of death cases, and clinical stage) between the training set and test set samples were observed, and they were found to be similar in the training set and test set (**Supplementary Figure 1**). Firstly, the univariate COX proportional hazard regression model was used to analyze each methylation site and survival data. When *p* < 0.05 was selected as the threshold, a total of 12,264 methylation sites significantly correlated with survival were obtained. Age (*p* = 0.0043) and tumor stage (*p* = 0.0012) were also significant factors. Age and grade were included in the COX proportional hazard regression model as covariates, and 13,739 methylation sites significantly correlated with survival were obtained, including 11,637 matching sites between the two analyses.

### Consensus Clustering of Glioma Identified Distinct DNA Methylation Prognosis Subgroups

**Figure 2:**
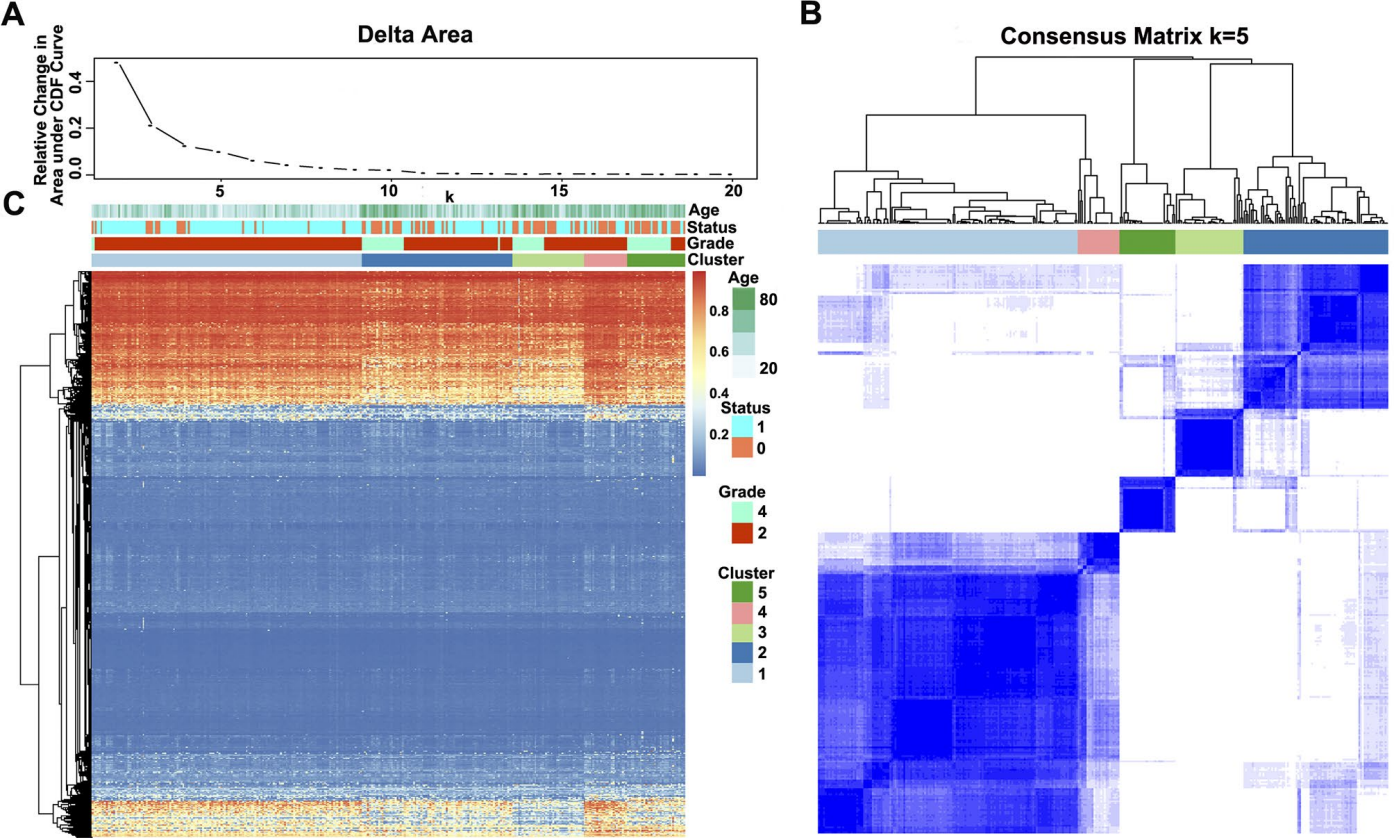
Consensus matrix for DNA methylation classification with the corresponding heat map. **(A)** Delta area curve of consensus clustering, indicating the relative change in area under the cumulative distribution function (CDF) curve for each category number ***k*** compared with ***k*** − 1. The horizontal axis represents the category number ***k***, and the vertical axis represents the relative change in area under the CDF curve. **(B)** Color-coded heat map corresponding to the consensus matrix for ***k*** = 5 obtained by applying consensus clustering. The color gradients were from 0 to 1, representing the degree of consensus, with white corresponding to 0 and dark blue to 1. **(C)** Heat map corresponding to the dendrogram in (B), which was generated using the pheatmap function with DNA methylation classification, tumor stage, age, and prognostic status as the annotations.

The methylation profiles of the 11,637 CpG sites from the 327 samples in the training set were employed for the consensus clustering of samples using the ConsensusClusterPlus *R* software package to obtain the glioma molecular subtypes. To determine the appropriate cluster number, we calculated the average cluster consistency and inter-cluster variation coefficient for the number of each cluster, respectively. Typically, the area under the CDF curve tended to be stable after five clusters (**Figure 2A**), the smallest variation coefficient among all clusters was 0.076, and the sample cluster number was 5 (**Supplementary Table 1**). Therefore, five was selected as a suitable cluster number for further analysis in this study (**Figure 2B**).

Notably, most methylation sites displayed low DNA methylation levels in each sample; additionally, there were also differences in the DNA methylation profile among the five clusters, and the DNA methylation levels of Cluster2, Cluster3, and Cluster5 were lower than those of Cluster1 and Cluster4 (**Figure 2C**).

Indeed, the methylation levels of these five subgroups were significantly related to some molecular genetic features. For example, the methylation levels were positively associated with TP53 mutant but were negatively associated with co-deletion of 1p/19q in Cluster1 (**Supplementary Table 2**). In Cluster2, tumor protein p53 (TP53) mutant, isocitrate dehydrogenase [NADP(+)] 1 (IDH1) mutant, and co-deletion of 1p/19q have been reported to be negatively associated with methylation levels (**Supplementary Table 3**). The methylation levels were positively related to O-6-methylguanine-DNA methyltransferase (MGMT) promoter unmethylation but were negatively associated with TP53 mutant, α-thalassemia mental retardation X-linked (ATRX) mutant, and co-deletion of 1p/19q in Cluster3 (**Supplementary Table 4**). In Cluster4, the methylation levels have been associated with IDH1 mutant, ATRX mutant, and *MGMT* promoter unmethylation (**Supplementary Table 5**). TP53 mutant, telomerase reverse transcriptase (TERT) mutant, and *MGMT* promoter unmethylation were associated with methylation levels in Cluster5 (**Supplementary Table 6**). Thus, the five subgroups based on the methylation levels may reflect changes in some molecular genetic features.

### Characterizing Different Characteristics of DNA Methylation Clustering

**Figure 3:**
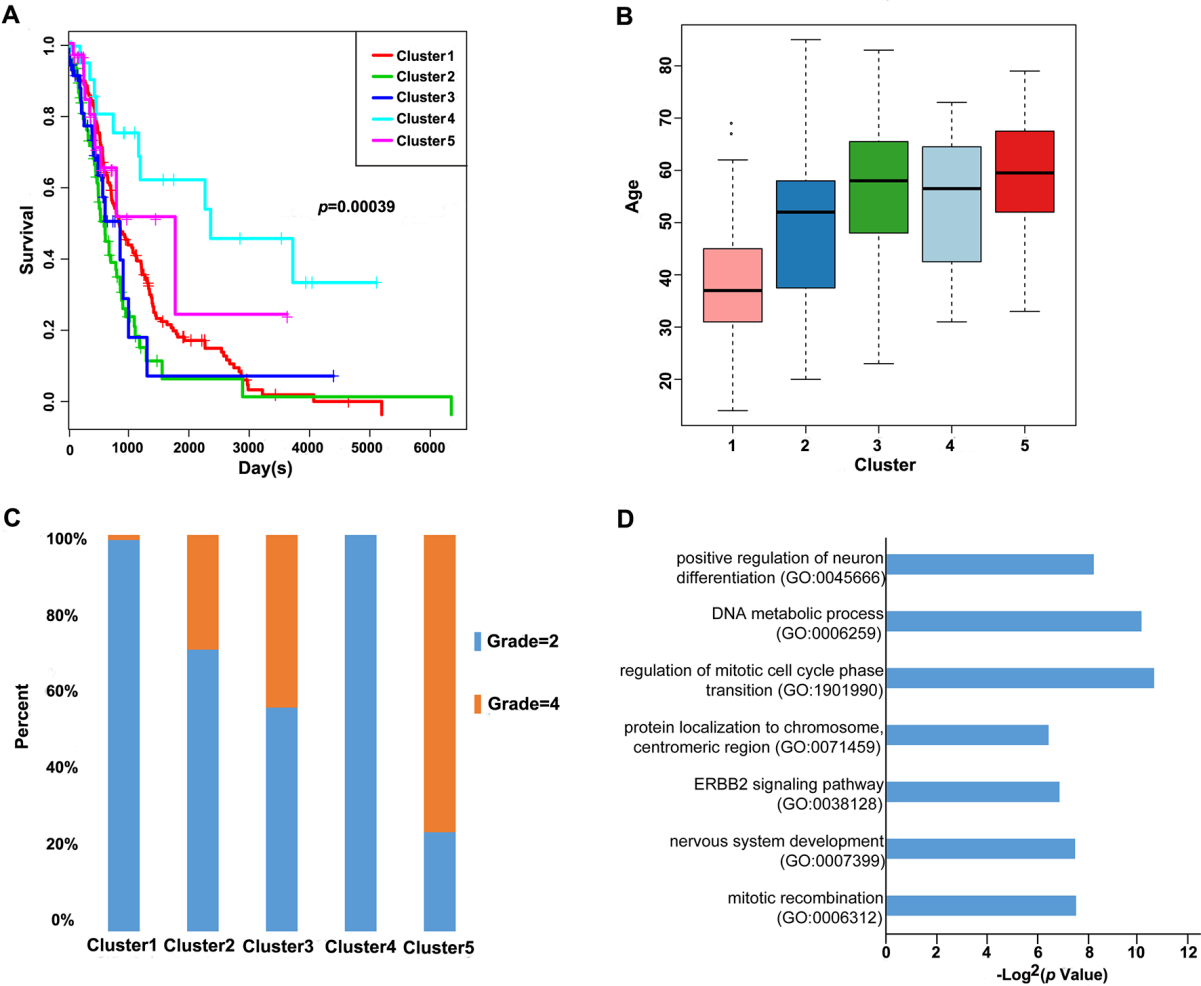
Prognosis, grade, age distribution, and survival of each sample in the molecular subtypes. **(A)** Survival curves of DNA methylation subtypes in the training set. The horizontal axis represents the survival time (days), and the vertical axis represents the probability of survival. The numbers in parentheses in the legend represent the number of samples in each cluster. The log-rank test was used to assess the statistical significance of the differences. **(B)** Age distributions of nine DNA methylation clusters in the training set. The horizontal axis represents the DNA methylation clustering. **(C)** Grade distributions of nine DNA methylation clusters in the training set. The horizontal axis represents the DNA methylation clustering. **(D)** The online network tool Enrichr was utilized for functional enrichment analysis of genes corresponding to the gene promoter regions annotated by the CpG sites that were significantly correlated with survival.

Furthermore, we analyzed the prognosis, grade and age distribution, and survival of each sample in the five molecular subtypes. It was discovered through Kaplan–Meier and log-rank tests that there were significant differences in prognosis among samples of these five molecular subtypes (*p* = 0.00039) (**Figure 3A**); Cluster4 had favorable prognosis, while Cluster2 and Cluster3 were associated with poor prognosis and relatively lower DNA methylation levels, revealing that the prognosis for low-methylated samples was poorer than that for highly methylated samples. It was also noted that patients in Cluster1 were generally between 30 and 45 years of age (**Figure 3B**) and were younger than patients in the other clusters. Comparing the tumor grades of the subgroups, 98.7% and 100% of the samples in Cluster1 and Cluster4 corresponded to glioma grade 2, respectively, while 71.1%, 56.4%, and 25% of the samples in Cluster2, Cluster3, and Cluster5 corresponded to grade 2, respectively (**Figure 3C**). Taken together, these results indicated that these DNA methylation sites could serve as important markers for prognosis.

**Figure 4:**
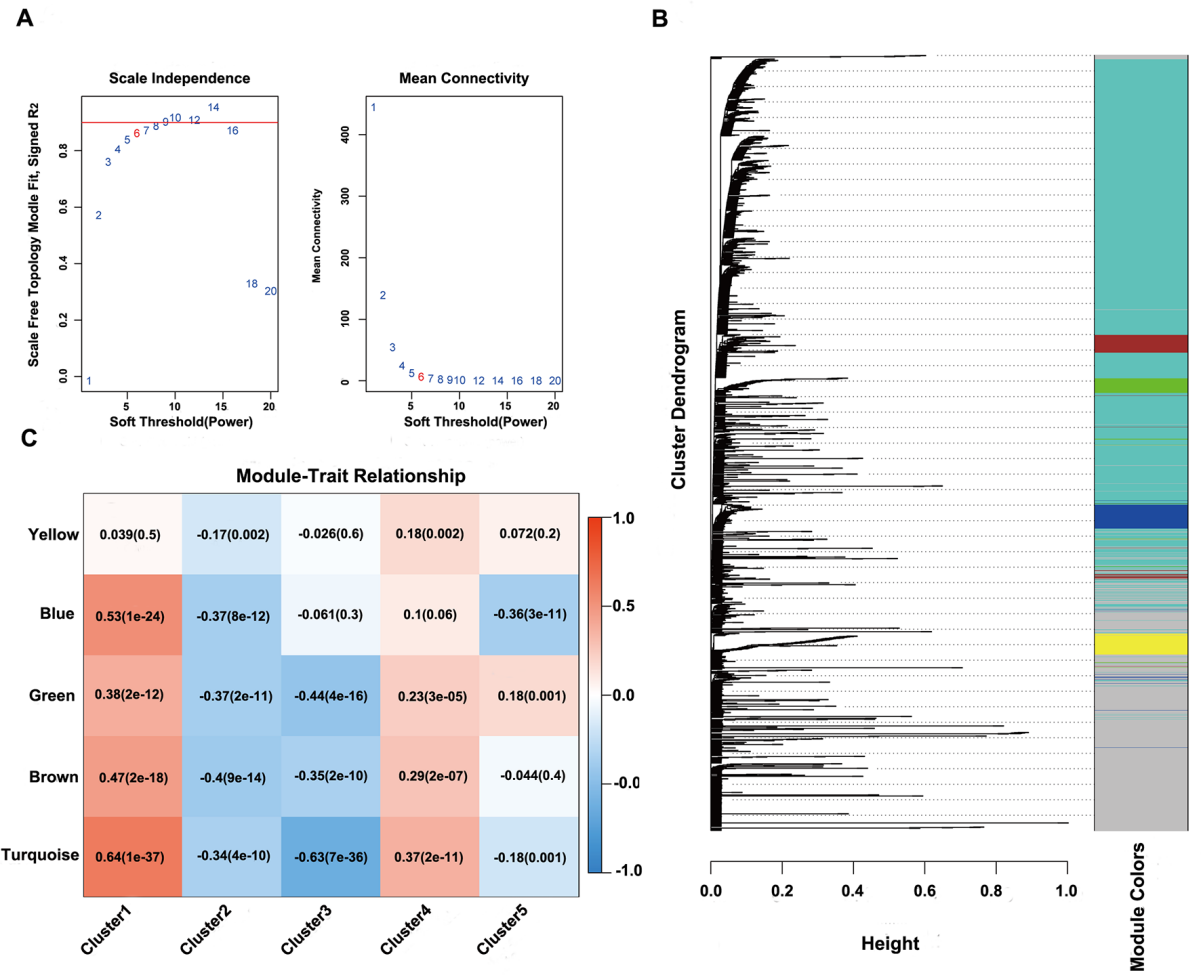
WGCNA analysis of CpG sites. **(A)** Scale-free topology index and mean connectivity were used to determine the soft threshold (â = 6). **(B)**, Clustering dendrogram of CpG sites. The dissimilarity of CpG sites is based on topological overlap. The genes are assigned to different modules and are identified using different colors. **(C)** Module–trait correlation analysis showed that five modules were significantly correlated with each cluster.

Next, the online network tool Enrichr was utilized for functional enrichment analysis of genes corresponding to the gene promoter regions annotated by the CpG sites that were significantly correlated with survival (Chen et al., 2013). It was found that these genes were enriched in the biological processes related to glioma, which included basic cancer-related biological processes, as well as glioma-related specific biological processes, including mitotic recombination, DNA metabolism, and ErbB2 signaling pathway (**Figure 3D**), suggesting that the methylation sites revealed in this study might affect gliomagenesis and development. The weight co-expression network was constructed using the weighted gene co-expression network analysis (WGCNA) *R* software package (Langfelder and Horvath, 2008), and to guarantee that the network was scale-free, the soft threshold â = 6 was selected (**Figure 4A**). Five modules were obtained after further analysis (**Figure 4B**), among which the gene numbers included in each module were 80, 67, 52, 637, 1,319, and 59, respectively (**Supplementary Table 7**). Analysis of the module–trait relationship showed that several of the modules displayed significant correlation or anti-correlation with the five glioma molecular subtypes (**Figure 4C**).

### Identifying Specific DNA Methylation Markers

Cluster4 was linked to the best prognosis among all clusters; therefore, all CpG sites in the turquoise module that was most correlated with Cluster4 were selected. The CpG sites (connectivity > 1000) in the network were selected as the feature methylation sites of Cluster4 samples, and the correlation among 108 CpG loci was significantly higher than that among other loci using Pearson correlation analysis. Ultimately, we chose 11 CpG loci, which intersected the 2 loci (**Supplementary Figure 2** and **Supplementary Table 8**).

### Constructing and Evaluating the Prognosis Prediction Model

**Figure 5:**
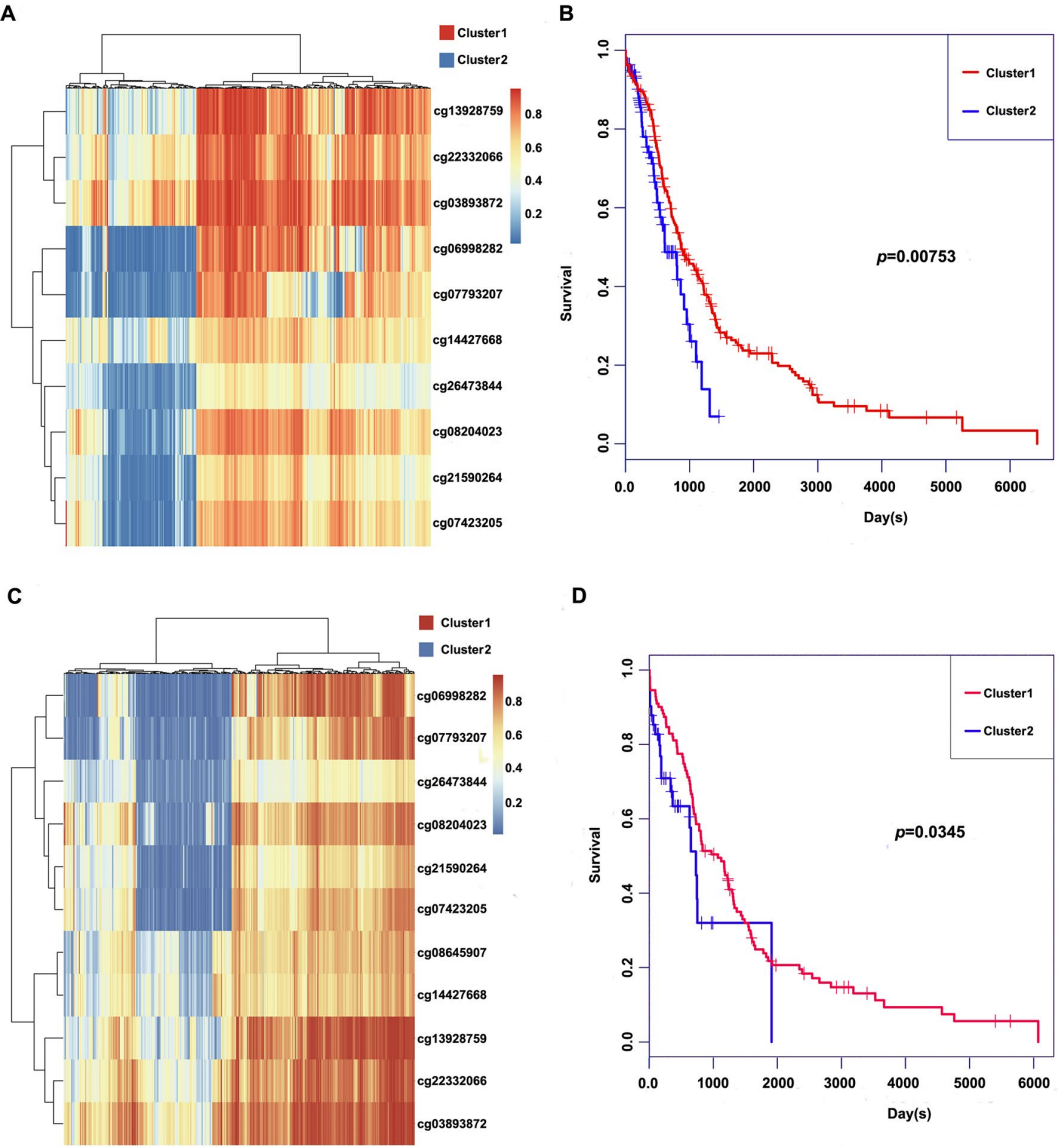
Clustering and survival results of the 11 CpG sites in the training and test set. **(A)** Consensus clustering of the 11 CpG sites in the training set. **(B)** Survival curves of two clusters predicted from the training set using the prognosis model. The log-rank test was used to assess the statistical significance of the difference. **(C)** Consensus clustering of the 11 CpG sites in the test set. **(D)** Survival curves of two clusters predicted from the test set using the prognosis model. The log-rank test was used to assess the statistical significance of the difference.

These 11 CpG methylation profiles were selected for further unsupervised cluster analysis; the similarity between samples was calculated by the Euclidean distance. The results suggested that the methylation levels of these 11 CpG sites could divide the samples into two groups, namely, Cluster1 and Cluster2, of which Cluster2 was the high-methylation group, while Cluster1 was the low-methylation group (**Figure 5A**). The difference in prognosis between the two groups was further analyzed, which revealed that the prognosis in the high-methylation group was worse than that in the low-methylation group (**Figure 5B**). The methylation profiles of these 11 CpG sites were extracted from the methylation profiles in the test set for further hierarchical cluster analysis. It was observed that the methylation profiles of these 11 CpG methylation sites could be clearly grouped into two clusters, among which the methylation level in Cluster1 samples was markedly lower than that in Cluster2 samples (**Figure 5C**). The distinct high-methylation and low-methylation samples were selected for survival analysis and demonstrated that the prognosis in highly methylated samples was notably worse than that in low-methylated samples (**Figure 5D**), which was consistent with the training set results.

**Figure 6:**
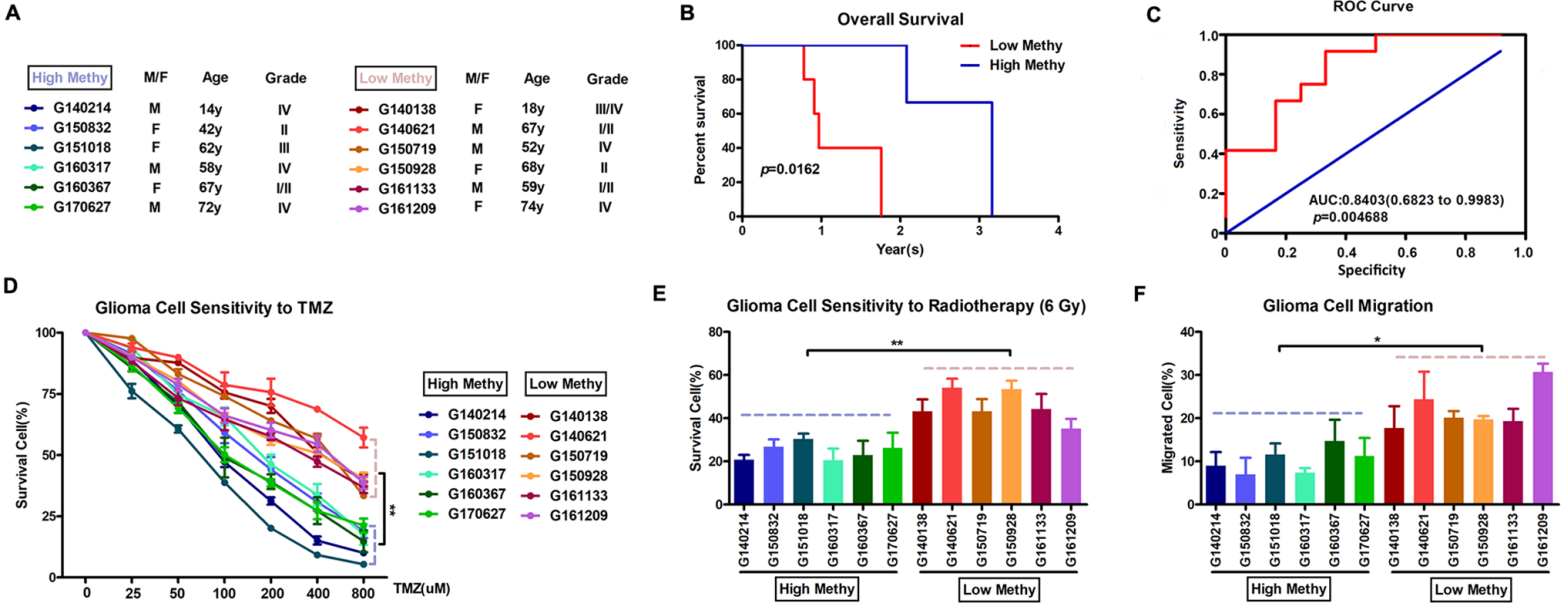
Application in clinical practice of the final prognostic predictor on 11 feature genes. **(A)** The clinical characteristics of the 12 glioma patients. **(B)** Survival curves of two clusters predicted from 12 glioma patients using the prognosis model. The log-rank test was used to assess the statistical significance of the difference. The red line indicates the low-methylation group (high-risk group), while the blue line indicates the high-methylation group (low-risk group), based on the final prognostic predictor. **(C)** receiveroperating characteristic (ROC) curve with AUC under the final prognostic predictor. **(D)** The proportion of surviving glioma cells derived from glioma patients after treatment with temozolomide with indicated concentration. **(E)** The proportion of surviving glioma cells derived from glioma patients after 6 Gy of irradiation. **(F)** The proportion of migrated glioma cells derived from glioma patients.

Based on the final prognostic predictor, we analyzed the clinical follow-up data of these 12 glioma patients, which were divided into the high-methylation group (n = 6) and low group (n = 6) (**Supplementary Figure 3** and **Figure 6A**). There was a positive correlation between the methylation level and overall survival (*p* = 0.0162) (**Figure 6B**), with an area under curve (AUC) of 0.8542 (**Figure 6C**). Consistent with these, there was an inverse relationship between the methylation level and insensitivity to temozolomide (or radiotherapy) (**Figures 6D, E**) or migration ability (**Figure 6F**) of glioma cells derived from GBM patients. Thus, we concluded that this prognostic predictor showed great promise for application in clinical practice.

## Discussion

Aberrant DNA methylation is one of the hallmarks of cancer tissues (Klutstein et al., 2016; Witt et al., 2018). Recent developments in sequencing technologies have made it possible to analyze genome-wide DNA methylation profiles at high resolution. Whole genome bisulfate sequencing is the best method to investigate DNA methylation; its efficacy, however, is limited by high analytic burden and cost. DNA methylation arrays are a good alternative for investigating genome-wide DNA methylation in a large collection of tumors. The TCGA database is a publicly available resource that covers a wide variety of data types in a variety of cancers; thus, the large sample sizes allowed us to explore glioma molecular subtypes more comprehensively.

Global loss of methylation and gene-specific DNA promoter methylation occur frequently during carcinogenesis, and these methylation alterations have been regarded as potential molecular markers for cancer initiation and progression (Dor and Cedar, 2018; Koch et al., 2018). DNA methylation in mammals mostly occurs at position 5′ of the cytosine ring in CpGs through a covalent bond of the methyl group (Arber and Linn, 1969; Yarus, 1969). Non-CpG sequences can also get methylated but with less frequency. In normal tissue, CpG island methylation usually increases with age, although the total genomic content of methylcytosine decreases (Perez et al., 2018). During carcinogenesis, a global loss of DNA methylation, together with tumor suppressor gene silencing by promoter DNA methylation, has been observed in most tumor types. Promoter methylation in tumor suppressor gene CpG islands has been demonstrated as a hallmark of cancer. Earlier research has profiled gene-specific promoter methylation in neck squamous cell carcinoma and head, bladder, lung, and liver cancers, among others.

Molecular mechanistic study based on bioinformatics analysis is a significant method in cancer research. Previous studies indicated that glioma could be classified into three groups based on patterns of global DNA methylation: glioma CpG island methylator phenotype (G-CIMP) (highly methylated), intermediately methylated, or low-methylated tumors (Verhaak et al., 2010). One problem associated with the use of clustering algorithms to classify tumors into subgroups is the failure to realize the “true” number of subgroups that are present in a data set. Here, we explored specific prognosis subtypes based on DNA methylation status using 653 gliomas from the TCGA database. To determine the appropriate cluster number, we calculated the average cluster consistency and inter-cluster variation coefficient for the number of each cluster, respectively. Typically, the area under the CDF curve tended to be stable after five clusters, the smallest variation coefficient among all clusters was 0.076, and the sample cluster number was 5. Thus, five subgroups were distinguished by consensus clustering using 11,637 CpGs that significantly influenced survival. Similar to recent studies (Ceccarelli et al., 2016; De Souza et al., 2018), the subgroups based DNA methylation was associated with patient age, advanced stage, and prognosis. Importantly, the methylation levels of different subgroups could reflect different molecular genetic features.

Multifold molecular analyses have been used to take advantage of tumor biology in response to prediction or risk stratification (Krajewska et al., 2017; Masci, 2017). It is known that transcriptional activity is regulated by methylation of cytosine residues, which constitutes a rather stable DNA modification. Reports on DNA methylation signature, which predicts cancer risk, are rare, however. It is important to discover tumor-specific prognostic factors for glioma to predict outcome and improve treatments. Here, WGCNA analysis of the CpG sites revealed that 11 of them could distinguish the samples into high- and low-methylation groups and could classify the prognostic information of samples after cluster analysis of the training set samples using the hierarchical clustering algorithm. It is worth noting that four CpG sites were found in the glial cell line–derived neurotrophic factor (GDNF) gene, a member of the transforming growth factor-â (TGF-â) superfamily, which signals *via* the tyrosine kinase receptor c-Ret and the Glial cell line-derived neurotrophic factor receptor(GDNF)-alpha (GFRá); meanwhile, it is well documented that GDNF also supports neuronal differentiation and dopaminergic development. Limited availability of clinical data and fresh tumor specimens symbolizing transitional steps from tumor initiation to progression is an important barrier to improving the clinical outcomes and therapeutic strategies for glioma patients. Now, we could analyze epigenomic profiles to understand the epigenome-based evolution of gliomas. At first recurrence, the IDH-wild-type stem cell–like GBM phenotype by G-CIMP-low showed molecular similarity to glial cell differentiation (De Souza et al., 2018). In our study, we found a series of CpG sites at genes involved in brain development or neuronal differentiation. These results could provide clues to the mechanism of the evolution of glioma. Indeed, genes involved in brain development and neuronal differentiation were strongly enriched among genes frequently methylated in tumors, for example, choline O-acetyltransferase (CHAT), GS homeobox 2 (GSX2), NK6 homeobox 1 (NKX6-1), paired box 6 (PAX6), retina and anterior neural fold homeobox (RAX) and distal-less homeobox 2 (DLX2) (Wu et al., 2010; Yu et al., 2013). The methylation of the genes involved in neuronal differentiation, in cooperation with other oncogenic events, may shift the balance from regulated differentiation towards gliomagenesis.

A recent report emphasized the relevance of DNA methylation profiles in somatic TERT pathway alterations (Ceccarelli et al., 2016). Indeed, functional enrichment analysis by Enrichr in our study found that these genes were enriched in the basic cancer-related biological processes, including mitotic recombination, DNA metabolism, and ErbB2 signaling pathway. These biological processes were significantly associated with telomere maintenance. Based on the final prognostic predictor, we analyzed the clinical follow-up data of these 12 glioma patients and found a positive correlation between methylation level and overall survival. Using *invitro* experiments, we also confirmed that glioma cells with low methylation level would have higher migration ability and show resistance to temozolomide (or radiotherapy) compared to cells with high methylation level. Thus, these results suggested that the model constructed in this study could provide guidance for clinicians regarding the prognosis of various epigenetic subtypes.

## Conclusion

Our research identified five different prognosis subgroups using glioma data in TCGA that differed either at the molecular level or in epidemiology, providing a more detailed explanation for glioma heterogeneousness. Additionally, our criteria will provide more targets for glioma precision medicine by identifying specific molecular markers for each subtype. Changes in DNA methylation can be used as markers to diagnose special subgroups, and clinicians can develop personalized treatments following these prognoses. Our approaches can also be used to study other tumors.

## Data Availability

Publicly available datasets were analyzed in this study. This data can be found here: https://cancergenome.nih.gov/

## Ethics Statement

The protocol of this article was approved by the Institutional Review Board of the Hefei Institutes of Physical Science, Chinese Academy of Sciences.

## Author Contributions

XC and ZF: conceived and designed the experiments. CZ and ZZ: collected the data. XC and CZ: performed the analysis. XC, HW, and ZF: participated in the discussion of the algorithm. XC and CZ: prepared and edited the manuscript. All authors have read and approved the final manuscript.

## Funding

This research was supported by the National Natural Science Foundation of China (81872066, 31571433, and 81773131), the innovative program of the Development Foundation of Hefei Center for Physical Science and Technology (2018CXFX004 and 2017FXCX008), and the Youth Innovation Promotion Association of the Chinese Academy of Sciences (2018487).

## Conflict of Interest Statement

The authors declare that the research was conducted in the absence of any commercial or financial relationships that could be construed as a potential conflict of interest.

## Abbreviations

CDF, consensus cumulative distribution function; CpG, cytosine preceding a guanosine; GBM, glioblastoma multiforme; knn, k-nearest neighbors; LGG, lower-grade glioma; SD, standard deviation; TCGA, The Cancer Genome Atlas; WGCNA, weighted gene co-expression network analysis.
