# Supplementary material for: Specific Glioma Prognostic Subtype Distinctions Based on DNA Methylation Patterns
Source: Front Genet. 2019 Sep 12;10:786. doi: 10.3389/fgene.2019.00786 (PMC6751377; doi:10.3389/fgene.2019.00786)
Supplement: Supplementary file 1 [file DataSheet_1.pdf]

Supplementary Table 1. The Number of Samples in the 5 Cluster.

Cluster	Number of Samples
Cluster1	149
Cluster2	83
Cluster3	39
Cluster4	24
Cluster5	32

Supplementary Table 2. Relationship between Gene Status and DNA Methylation Level in Cluster1.

	Gene Status	Pearson	*p* Value
1	TP53 （Mutant）	0.17	3.791e-3
2	IDH1 （Mutant）	-0.04	0.525
3	MGMT （Unmethylated）	0.01	0.932
4	1p/19q （Non-codeletion）	-0.22	2.266e-4
5	TERT （Mutant）	0.06	0.345
6	ATRX （Mutant）	-0.06	0.352

Supplementary Table 3. Relationship between Gene Status and DNA Methylation Level in Cluster2.

	Gene Status	Pearson	*p* Value
1	TP53 （Mutant）	-0.524	7.17e-8
2	IDH1 （Mutant）	-0.365	5.094e-4
3	MGMT （Unmethylated）	0.157	0.197
4	1p/19q （Non-codeletion）	-0.196	0.0243
5	TERT （Mutant）	-0.0811	0.377
6	ATRX （Mutant）	0.0795	0.562

Supplementary Table 4. Relationship between Gene Status and DNA Methylation Level in Cluster3.

	Gene Status	Pearson	*p* Value
1	TP53（Mutant）	-0.362	5.708e-4
2	IDH1（Mutant）	-0.147	0.224
3	MGMT（Unmethylated）	0.157	1.561e-3
4	1p/19q（Non-codeletion）	-0.114	0.00123
5	TERT（Mutant）	0.0811	0.551
6	ATRX（Mutant）	-0.0416	0.001786

Supplementary Table 5. Relationship between Gene Status and DNA Methylation Level in Cluster4.

	Gene Status	Pearson	*p* Value
1	TP53 （Mutant）	0.0692	0.620
2	IDH1 （Mutant）	-0.0967	1.1013e-3
3	MGMT （Unmethylated）	0.403	8.865e-5
4	1p/19q （Non-codeletion）	0.0561	0.0722
5	TERT （Mutant）	-0.0489	3.426e-6
6	ATRX （Mutant）	0.409	0.0562

Supplementary Table 6. Relationship between Gene Status and DNA Methylation Level in Cluster5.

	Gene Status	Pearson	*p* Value
1	TP53 （Mutant）	-0.0678	3.62e-4
2	IDH1 （Mutant）	0.0563	0.0685
3	MGMT （Unmethylated）	0.0409	1.932e-4
4	1p/19q （Non-codeletion）	0.0342	0.0781
5	TERT （Mutant）	-0.762	4.67e-2
6	ATRX （Mutant）	0.0352	0.782

Supplementary Table 7. The Number of Genes in the 7 Modules.

Module Colors	Freq
Blue	80
Brown	67
Green	52
Grey	637
Turquoise	1319
Yellow	59

Supplementary Table 8. The Annotationof 11 CpG Sites.

CpG	Chromosome	Site	GeneSymbol
cg08204023	5	37835109	GDNF
cg26473844	5	37834909	GDNF
cg06998282	8	1.21E+08	ENPP2
cg21590264	5	37834850	GDNF
cg07423205	5	37834672	GDNF
cg22332066	1	2.11E+08	HHAT
cg14427668	7	1.05E+08	ATXN7L1
cg07793207	10	95517529	LGI1
cg03893872	1	2.11E+08	HHAT
cg08645907	7	105319679	ATXN7L1
cg13928759	10	95517382	LGI1

Supplementary Figure 1

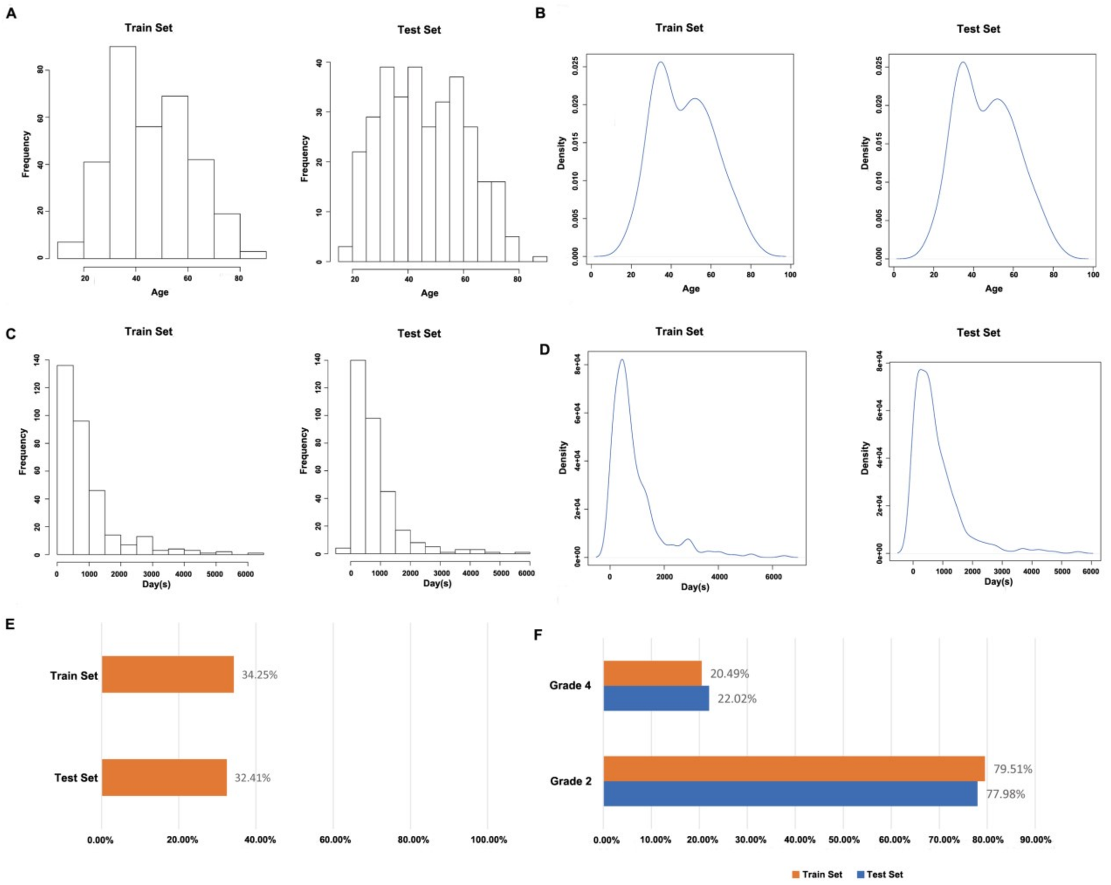

Supplementary Figure 1. Comparisons of Four Properties between Training Set and Test Set Samples. The 653 samples were evenly divided into a training set (n=327) and test set (n=326), and four properties including age (**A**, **B**), follow-up period (**C**, **D**), proportion of death samples (**E**), and tumor grade (**F**) were found to be similar in the training set and test set.

Supplementary Figure 2

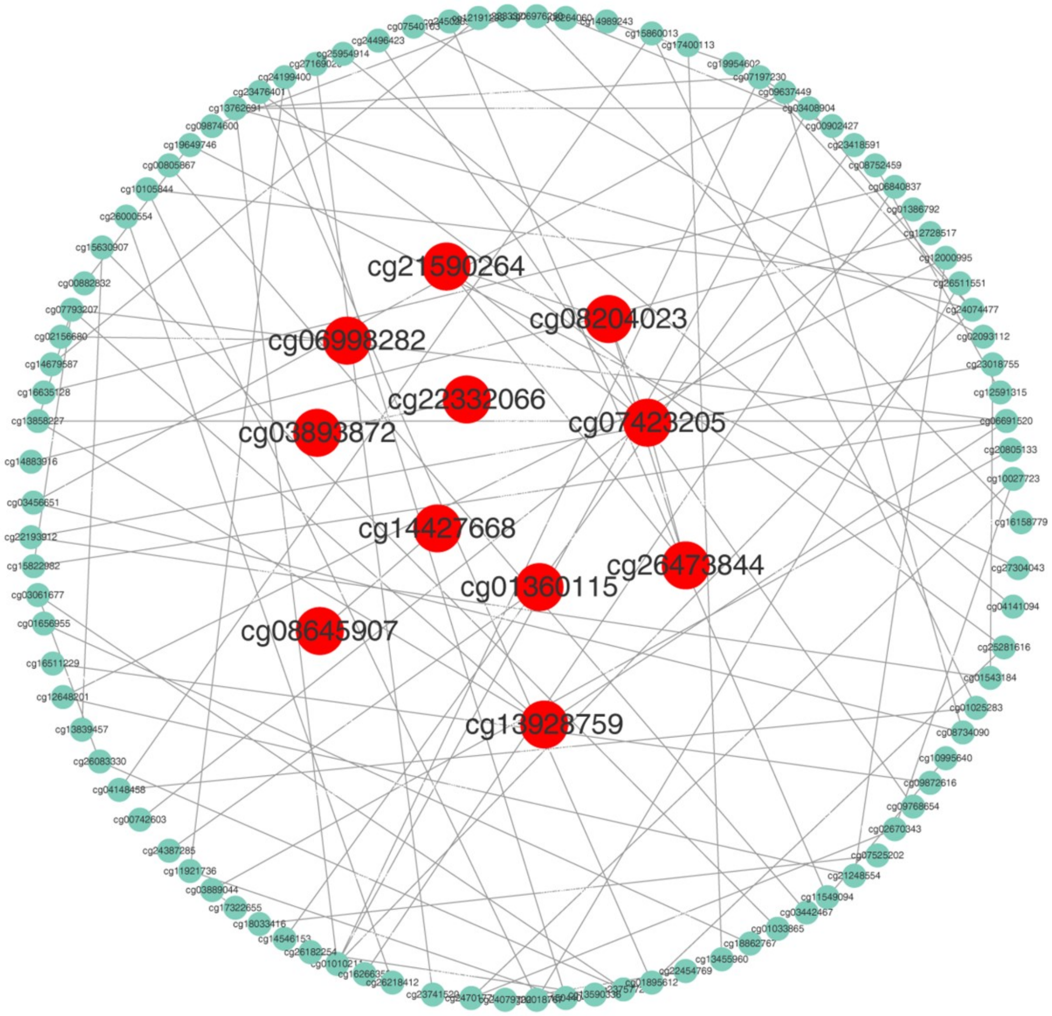

Supplementary Figure 2. Relationship Network of the Feature Methylation Sites.

Supplementary Figure 3

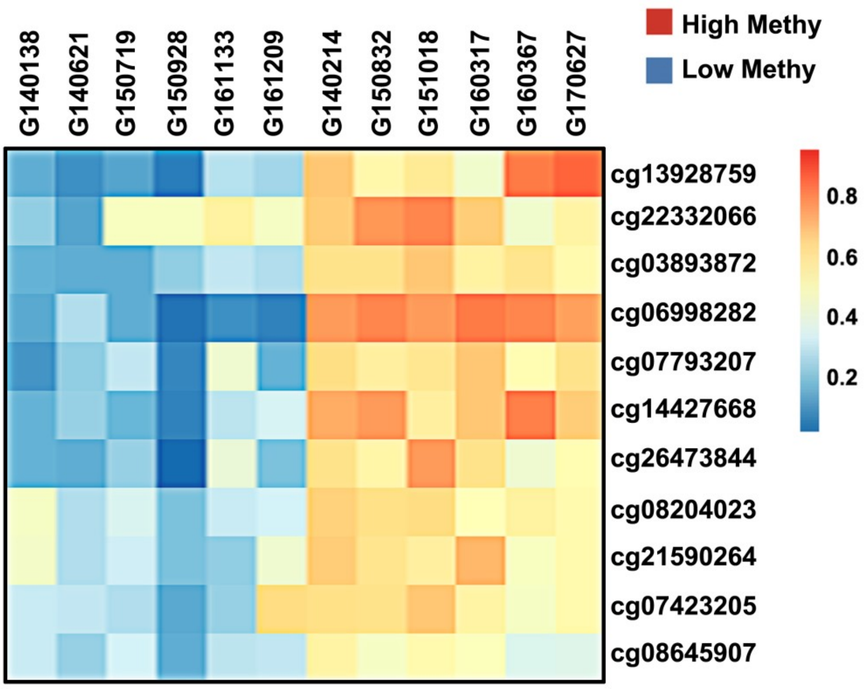

Supplementary Figure 3. Consensus Clustering of the 11 CpG Sites of 12 glioma Patients.
