# Supplementary material for: Specific Glioma Prognostic Subtype Distinctions Based on DNA Methylation Patterns
Source: Front Genet. 2019 Sep 12;10:786. doi: 10.3389/fgene.2019.00786 (PMC6751377; doi:10.3389/fgene.2019.00786)
Supplement: Supplementary file 2 [file Table_1.doc]

**Title**

Specific Glioma Prognostic-subtype Distinctions Based on DNA Methylation Patterns

**Running title**

Glioma Prognosis-subtype Distinctions

Xueran Chen ^a, b, *^, Chenggang Zhao ^a, c^, Zhiyang Zhao ^a, c^, Hongzhi Wang ^a, b^, and Zhiyou Fang ^a, b, #^

^a^ Anhui Province Key Laboratory of Medical Physics and Technology; Center of Medical Physics and Technology, Hefei Institutes of Physical Science, Chinese Academy of Sciences, No. 350, Shushan Hu Road, Hefei, Anhui, 230031, China

^b^ Hefei Cancer Hospital, Chinese Academy of Sciences, No. 350, Shushan Hu Road, Hefei, Anhui, 230031, China

^c^ University of Science and Technology of China, No. 96, Jin Zhai Road, Hefei, Anhui, 230026, China

*Corresponding author: Dr. Xueran Chen. Anhui Province Key Laboratory of Medical Physics and Technology; Center of Medical Physics and Technology, Hefei Institutes of Physical Science, Chinese Academy of Sciences, No. 350, Shushan Hu Road, Hefei, Anhui, 230031, China. Telephone/fax: 86-551-62796705, e-mail: xueranchen@cmpt.ac.cn

^#^Corresponding author: Prof. Zhiyou Fang. Anhui Province Key Laboratory of Medical Physics and Technology; Center of Medical Physics and Technology, Hefei Institutes of Physical Science, Chinese Academy of Sciences, No. 350, Shushan Hu Road, Hefei, Anhui, 230031, China. Telephone/fax: 86-551-62727067, e-mail: [z.fang@cmpt.ac.cn](mailto:z.fang@cmpt.ac.cn)

**Author email addresses:**

Dr. Xuern Chen: xueranchen@cmpt.ac.cn

Mr. Chenggang Zhao: [352782963@qq.com](mailto:352782963@qq.com)

Mr. Zhiyang Zhao: [zzhiyang@mail.ustc.edu.cn](mailto:zzhiyang@mail.ustc.edu.cn)

Pro. Hongzhi Wang: [wanghz@hfcas.ac.cn](mailto:wanghz@hfcas.ac.cn)

Pro. Zhiyou Fang: [z.fang@cmpt.ac.cn](mailto:z.fang@cmpt.ac.cn)

**Abstract**

DNA methylation is an important regulator of gene expression and may provide an important basis for effective glioma diagnosis and therapy. Here, we explored specific prognosis‐subtypes based on DNA methylation status using 653 gliomas from the TCGA database. Five subgroups were distinguished by consensus clustering using 11,637 CpGs that significantly influenced survival. The specific DNA methylation patterns were correlated with age, tumor stage, and prognosis. Additionally, WGCNA analysis of CpG sites revealed that 11 of them could distinguish the samples into high- and low-methylation groups and could classify the prognostic information of samples after cluster analysis of the training set samples using the hierarchical clustering algorithm. Similar results were obtained from the test set and 12 glioma patients. Moreover, *in vitro* experiments revealed an inverse relationship between methylation level and migration ability or insensitivity to temozolomide (or radiotherapy) of glioma cells based on the final prognostic predictor. Thus, these results suggested that the model constructed in this study could provide guidance for clinicians regarding the prognosis of various epigenetic subtypes.

Keywords: Glioma; Consensus Clustering; DNA Methylation; Molecular Subtypes; Prognosis

1. **Introduction**

Glioma derives from glial cells and is the most prevalent primary central nervous system malignant tumor . The overall survival time continues to be unsatisfactory, especially for high-grade glioma, although treatment strategies, including surgical resection, radiation, and chemotherapy, for glioma patients have been greatly improved . It is therefore urgent to elucidate the molecular mechanisms underlying glioma tumorigenesis for developing novel therapies.

Epigenetics is recognized as heritable alterations in gene expression not connected to an alteration in DNA sequence, but plays a crucial role in carcinogenesis . Cancer epigenetics covers aspects of aberrant DNA methylation, dysregulated non- coding RNA, and altered post-translational histone modification, among which aberrant DNA methylation is most widely investigated . Aberrant DNA methylation could influence the key genes that are involved in glioma carcinogenesis and progression and may especially influence some tumor suppressor genes by altering their expression and inhibiting their function . Thus, biological processes, specifically alterations in DNA methylation, can provide an important basis for early diagnosis and prognosis of cancer and development of new approaches for further clinical applications. Although the effects of certain genes with aberrant DNA methylation on glioma have been reported extensively, the comprehensive profile of the interaction network still needs further elucidation.

During the last decades, bioinformatics analysis and microarray technology have been widely used to identify general genetic or epigenetic alterations in carcinogenesis and screen biomarkers for prognosis and diagnosis of cancer . Several single genes whose global methylation status correlates with glioma outcome and gene expression level have already been identified . Additionally, some researches on aberrant DNA methylation have been conducted to identify glioma DNA methylation subtypes by DNA methylation profiles ; however, this classification was not detailed enough, and the specific sites that are associated with each category are unclear.

In this study, we addressed glioma classification by identifying specific prognosis-subtypes based on DNA methylation profiles of glioma obtained from The Cancer Genome Atlas (TCGA) database. This classification system may help identify molecular subtypes or new glioma markers to subdivide glioma patients more accurately. Moreover, our classification system provides guidance for clinicians on personalized treatments and diagnoses by identifying differences in prognosis for each epigenetic subtype.

**2. Materials and Methods**

### 2.1 Data Pre-processing and the Initial Screening of DNA Methylation Loci in Glioma

Lower grade glioma (LGG) and glioblastoma multiforme (GBM) DNA methylation data generated with the Illumina Infinium HumanMethylation450 BeadChip array were downloaded from the TCGA data portal . Methylation level of each probe was represented by the β‐value, which ranges from 0 to 1, corresponding to unmethylated and fully methylated, respectively. Probes with missing data in more than 70% of the samples were removed. The remaining probes with not available (NAs) were imputed using the k‐nearest neighbors (knn) imputation procedure. The ComBat algorithm in sva *R* package was used to remove batch effects by incorporating patient ID information and batch and integrating all the DNA methylation array data. Unstable genomic sites, including CpGs in single nucleotide polymorphisms and sex chromosomes, were removed. We selected CpGs in promoter regions, because DNA methylation in promoter regions influences gene expression strongly. Promoter regions were defined as 2 kb upstream to 0.5 kb downstream from transcription start sites. Finally, we selected samples having gene expression profiles. In total, 653 gliomas were used for the analysis.

Next, we separated the dataset into two cohorts: a training set and a test set. The criteria for this grouping were as follows: (a) random division of samples into two groups; (b) similar age distribution, staging, follow‐up time, and death ratio in the two groups.

**2.2 Determining Classification Features by COX Proportional Risk Regression Models**

CpG sites influencing survival significantly were used as classification features. First, univariate COX proportional risk regression models were constructed with methylation levels of each CpG site, age, and stage, and survival data of the cases. Then, the significant CpGs obtained from univariate COX proportional risk regression models were introduced into multivariate COX proportional risk regression models, using tumor stage and age as covariates, which were also significant in the univariate models. Finally, the CpG sites that were still significant were used as classification features. COX proportional hazard models were fitted with methylation levels of CpGs using the coxph function in survival package *R*, with clinical and demographic attributes (stage and age) as covariates in the multivariate analysis.

**2.3 Consensus Clustering to Obtain Molecular Subtypes Associated with Glioma Prognosis**

Consensus clustering was performed with the ConsensusClusterPlus package in *R* to determine subgroups of gliomas based on the most variable CpG sites . In this study, 80% of the samples were sampled 100 times by adopting the resampling program; the similarity distance between samples was estimated by the Euclidean distance , and kmdist was used as the clustering algorithm to search for the reliable and stable subgroup classification. After executing ConsensusClusterPlus, the item‐consensus results and cluster consensus were obtained. The criteria to determine the number of clusters were as follows: relatively high consistency within clusters, relatively low variation coefficient, and no appreciable rise in the area under the cumulative distribution function (CDF) curve. Variation coefficient was calculated according to the following formula: CV=(SD/MN)*100%, where MN represents the average of samples and SD represents the standard deviation. The category number was selected as the area under the CDF curve and showed no significant change. The heatmap corresponding to the consensus clustering was generated by pheatmap *R* package.

**2.4 Survival and Clinical Characteristics Analyses**

Kaplan-Meier plots were used to determine overall survival among glioma subgroups defined by DNA methylation profiles. The log‐rank test was used to measure the significance differences among the clusters. Survival analyses were performed with the survival package in *R* software. Associations between biological and clinical characteristics and DNA methylation clustering were analyzed with the chi‐square test. All tests were two‐sided and for all statistical tests, *p*<0.05 was considered to be significant unless otherwise noted.

**2.5 Glioma Cell Survival and Migration Assays**

### After receiving informed consent, glioma specimens were obtained from patients undergoing surgery at the Hefei Cancer Hospital, Chinese Academy of Sciences in accordance with the Institutional Review Boards. Within hours after surgical removal, tumor specimens were enzymatically dissociated into single cells, following previously reported procedures . For cell survival assay, the cells were plated at a seeding density of 10,000 cells/plate in a 60-mm plate, treated with or without temozolomide or 6 Gy radiotherapy, grew for 48 h in a standard growth medium, and washed with PBS. For cell migration assay, cell suspension in serum-free medium was added to the upper Transwell chamber, and then incubated for 18 h. The cells were fixed in cold methanol for 20 min, washed, and stored. Fixed cell colonies were visualized by incubating the cells with 0.5% (w/v) crystal violet for 0.5 h. Excess crystal violet was removed by washing with PBS. Cells that survived or migrated were counted. Differences in means were considered statistically significant when *p*<0.05 using a two-tailed *t* test.

**3. Results**

**3.1 DNA Methylation Features for Classification Based on Prognosis**

To identify the specific CpG sites that were significantly correlated with survival in glioma, we setup the workflow shown in Figure 1. The 450 k methylation profiles were downloaded from TCGA; 485,577 CpG sites in 685 samples and clinical follow-up information from 1,148 cases were obtained. There were 653 matched samples between clinical data and methylation profiles. The samples were evenly divided into a training set (n=327) and test set (n=326); four properties (including age, follow-up period, proportion of death cases, and clinical stage) between the training set and test set samples were observed, and they were found to be similar in the training set and test set (Supplementary Figure 1). Firstly, the univariate Cox proportional hazard regression model was used to analyze each methylation site and survival data. When *p*<0.05 was selected as the threshold, a total of 12,264 methylation sites significantly correlated with survival were obtained. Age (*p*=0.0043) and tumor stage (*p*=0.0012) were also significant factors. Age and grade were included in the Cox proportional hazard regression model as covariates, and 13,739 methylation sites significantly correlated with survival were obtained, including 11,637 matching sites between the two analyses.

**3.2 Consensus Clustering of Glioma Identified Distinct DNA Methylation Prognosis Subgroups**

The methylation profiles of the 11,637 CpG sites from the 327 samples in the training set were employed for the consensus clustering of samples using the ConsensusClusterPlus *R* software package to obtain the glioma molecular subtypes. To determine the appropriate cluster number, we calculated the average cluster consistency and inter-cluster variation coefficient for the number of each cluster, respectively. Typically, the area under the CDF curve tended to be stable after five clusters (Figure 2A), the smallest variation coefficient among all clusters was 0.076, and the sample cluster number was five (Supplementary Table 1). Therefore, five was selected as a suitable cluster number for further analysis in this study (Figure 2B).

Notably, most methylation sites displayed low DNA methylation levels in each sample; additionally, there were also differences in the DNA methylation profile among the five clusters, and the DNA methylation levels of Clusters2, 3, and 5 were lower than those of Cluster1 and Cluster4 (Figure 2C).

Indeed, the methylation levels of these five subgroups were significantly related to some molecular genetic features. For example, the methylation levels were positively associated with TP53 mutant, but were negatively associated with co-deletion of 1p/19q in Cluster1 (Supplementary Table 2). In Cluster2, TP53 mutant, IDH1 mutant, and co-deletion of 1p/19q have been reported to be negatively associated with methylation levels (Supplementary Table 3). The methylation levels were positively related to MGMT promoter unmethylation, but were negatively associated with TP53 mutant, ATRX mutant, and co-deletion of 1p/19q in Cluster3 (Supplementary Table 4). In Cluster4, the methylation levels have been associated with IDH1 mutant, ATRX mutant, and MGMT promoter unmethylation (Supplementary Table 5). TP53 mutant, TERT mutant, and MGMT promoter unmethylation were associated with methylation levels in Cluster5 (Supplementary Table 6). Thus, the five subgroups based on the methylation levels may reflect changes in some molecular genetic features.

**3.3 Characterizing Different Characteristics of DNA Methylation Clustering**

Furthermore, we analyzed the prognosis, grade and age distribution, and survival of each sample in the five molecular subtypes. It was discovered through Kaplan-Meier and log-rank tests that there were significant differences in prognosis among samples of these five molecular subtypes (*p*=0.00039) (Figure 3A); Cluster4 had favorable prognosis, while Clusters2 and 3 were associated with poor prognosis and relatively lower DNA methylation levels, revealing that the prognosis for low-methylated samples was poorer than that for highly methylated samples. It was also noted that patients in Cluster1 were generally between 30 and 45 years of age (Figure 3B) and were younger than patients in the other clusters. Comparing the tumor grades of the subgroups, 98.7% and 100% of the samples in Clusters1 and 4 corresponded to glioma grade 2, respectively, while 71.1%, 56.4%, and 25% of the samples in Clusters2, 3, and 5 corresponded to grade 2, respectively (Figure 3C). Taken together, these results indicated that these DNA methylation sites could serve as important markers for prognosis.

Next, the online network tool “Enrichr” was utilized for functional enrichment analysis of genes corresponding to the gene promoter regions annotated by the CpG sites that were significantly correlated with survival . It was found that these genes were enriched in the biological processes related to glioma, which included basic cancer-related biological processes, as well as glioma-related specific biological processes, including mitotic recombination, DNA metabolism, and ErbB2 signaling pathway (Figure 3D), suggesting that the methylation sites revealed in this study might affect gliomagenesis and development. The weight co-expression network was constructed using the WGCNA *R* software package , and to guarantee that the network was scale-free, the soft threshold â=6 was selected (Figure 4A). Five modules were obtained after further analysis (Figure 4B), among which, the gene numbers included in each module were 80, 67, 52, 637, 1319, and 59, respectively (Supplementary Table 7). Analysis of the module-trait relationship showed that several of the modules displayed significant correlation or anti-correlation with the 5 glioma molecular subtypes (Figure 4C)**.**

**3.4 Identifying Specific DNA Methylation Markers**

Cluster4 was linked to the best prognosis among all clusters; therefore, all CpG sites in the turquoise module that was most correlated with Cluster4 were selected. The CpG sites (connectivity>1000) in the network were selected as the feature methylation sites of Cluster4 samples, and the correlation among 108 CpG loci was significantly higher than that among other loci using Pearson correlation analysis. Ultimately, we chose 11 CpG loci, which intersected the two loci (Supplementary Figure 2 and Supplementary Table 8).

**3.5 Constructing and Evaluating the Prognosis Prediction Model**

These 11 CpG methylation profiles were selected for further unsupervised cluster analysis; the similarity between samples was calculated by the Euclidean distance. The results suggested that the methylation levels of these 11 CpG sites could divide the samples into two groups, namely, Cluster1 and 2, among which Cluster2 was the high methylation group, while Cluster1 was the low methylation group (Figure 5A). The difference in prognosis between two groups was further analyzed, which revealed that the prognosis in the high methylation group was worse than that in the low methylation group (Figure 5B). The methylation profiles of these 11 CpG sites were extracted from the methylation profiles in the test set for further hierarchical cluster analysis. It was observed that the methylation profiles of these 11 CpG methylation sites could be clearly grouped into two clusters, among which the methylation level in Cluster1 samples was markedly lower than that in Cluster2 samples (Figure 5C). The distinct high methylation and low methylation samples were selected for survival analysis and demonstrated that the prognosis in highly methylated samples was notably worse than that in lowly methylated samples (Figure 5D), which was consistent with the training set results.

### Based on the final prognostic predictor, we analyzed the clinical follow-up data of these 12 glioma patients, which were divided into the high methylation group (n=6) and low group (n=6) (Supplementary Figure 3 and Figure 6A). There was a positive correlation between the methylation level and overall survival (*p*=0.0162) (Figure 6B), with AUC of 0.8542 (Figure 6C). Consistent with these, there was an inverse relationship between the methylation level and insensitivity to temozolomide (or radiotherapy) (Figure 6 D and E) or migration ability (Figure 6F) of glioma cells derived from GBM patients. Thus, we concluded that this prognostic predictor showed great promise for application in clinical practice.

### 4. Discussion

Aberrant DNA methylation is one of the hallmarks of cancer tissues . Recent developments in sequencing technologies have made it possible to analyze genome-wide DNA methylation profiles at high resolution. Whole genome bisulfate sequencing is the best method to investigate DNA methylation; its efficacy, however, is limited by high analytic burden and cost. DNA methylation arrays are a good alternative for investigating genome-wide DNA methylation in a large collection of tumors. The TCGA database is a publicly available resource that covers a wide variety of data types in a variety of cancers; thus, the large sample sizes allowed us to explore glioma molecular subtypes more comprehensively.

Global loss of methylation and gene-specific DNA promoter methylation occur frequently during carcinogenesis, and these methylation alterations have been regarded as potential molecular markers for cancer initiation and progression . DNA methylation in mammals mostly occurs at position 5′ of the cytosine ring in CpGs through a covalent bond of the methyl group . Non-CpG sequences can also get methylated but with less frequency. In normal tissue, CpG island methylation usually increases with age, although the total genomic content of methylcytosine decreases . During carcinogenesis, a global loss of DNA methylation, together with tumor suppressor gene silencing by promoter DNA methylation, has been observed in most tumor types. Promoter methylation in tumor suppressor gene CpG islands has been demonstrated as a hallmark of cancer. Earlier researches have profiled gene-specific promoter methylation in neck squamous cell carcinoma and head, bladder, lung, and liver cancers, among others.

Molecular mechanistic study based on bioinformatics analysis is a significant method in cancer research. Previous studies indicated that glioma could be classified into three groups based on patterns of global DNA methylation; gCIMP (highly methylated), intermediately methylated, or low methylated tumors . One problem associated with the use of clustering algorithms to classify tumors into subgroups is the failure to realize the "true" number of subgroups that are present in a dataset. Here, we explored specific prognosis‐subtypes based on DNA methylation status using 653 gliomas from the TCGA database. To determine the appropriate cluster number, we calculated the average cluster consistency and inter-cluster variation coefficient for the number of each cluster, respectively. Typically, the area under the CDF curve tended to be stable after 5 clusters, the smallest variation coefficient among all clusters was 0.076, and the sample cluster number was 5. Thus, five subgroups were distinguished by consensus clustering using 11,637 CpGs that significantly influenced survival. Similar to recent studies , the subgroups based DNA methylation was associated with patient age, advanced stage and prognosis. Importantly, the methylation levels of different subgroups could reflect different molecular genetic features.

### Multifold molecular analyses have been used to take advantage of tumor biology in response to prediction or risk stratification . It is known that transcriptional activity is regulated by methylation of cytosine residues, which constitutes a rather stable DNA modification. Reports on DNA methylation signature, which predicts cancer risk, are however rare. It is important to discover tumor-specific prognostic factors for glioma to predict outcome and improve treatments. **Here, WGCNA analysis of the CpG sites revealed that 11 of them could distinguish the samples into high- and low-methylation groups and could classify the prognostic information of samples after cluster analysis of the training set samples using the hierarchical clustering algorithm.** It is worth noting that four CpG sites were found in the glial cell line-derived neurotrophic factor (GDNF) gene, a member of the TGF-â superfamily, which signals via the tyrosine kinase receptor c-Ret and the GDNF receptor GFRá; meanwhile, it is well documented that GDNF also supports neuronal differentiation and dopaminergic development. Limited availability of clinical data and fresh tumor specimens symbolizing transitional steps from tumor initiation to progression is an important barrier to improving the clinical outcomes and therapeutic strategies for glioma patients. Now, we could analyze epigenomic profiles to understand the epigenome-based evolution of gliomas. At first recurrence, the IDH-wild-type stem cell-like GBM phenotype by glioma-CpG island methylator phenotype (G-CIMP)-low showed molecular similarity to glia cell differentiation . In our study, we found a series of CpG sites at genes involved brain development or neuronal differentiation. These results could provide clues to the mechanism to the evolution of glioma. Indeed, genes involved in brain development and neuronal differentiation were strongly enriched among genes frequently methylated in tumors, for example CHAT, GSX2, NKX6-1, PAX6, RAX, and DLX2 . The methylation of the genes involved in neuronal differentiation, in cooperation with other oncogenic events, may shift the balance from regulated differentiation towards gliomagenesis.

### Recent report emphasized the relevance of DNA methylation profiles in somatic TERT pathway alterations . Indeed, functional enrichment analysis by “Enrichr” in our study was found that these genes were enriched in the biological processes related to basic cancer-related biological processes, including mitotic recombination, DNA metabolism, and ErbB2 signaling pathway. These biological processes were significantly associated with [telomere maintenance](https://www.sciencedirect.com/topics/biochemistry-genetics-and-molecular-biology/telomere-homeostasis). Based on the final prognostic predictor, we analyzed the clinical follow-up data of these 12 glioma patients, and found a positive correlation between methylation level and overall survival. Using *in vitro* experiments, we also confirmed that glioma cells with low methylation level would have higher migration ability and show resistance to temozolomide (or radiotherapy) compared to cells with high methylation level. Thus, these results **suggested that the model constructed in this study could provide guidance for clinicians regarding the prognosis of various epigenetic subtypes.**

### Conclusion

Our research identified five different prognosis-subgroups using glioma data in TCGA different at either the molecular level or in epidemiology, providing a more detailed explanation for glioma heterogeneousness. Additionally, our criteria will provide more targets for glioma precision medicine by identifying specific molecular markers for each subtype. Changes in DNA methylation can be used as markers to diagnose special subgroups, and clinicians can develop personalized treatments following these prognoses. Our approaches can also be used to study other tumors.

**Abbreviations**

CDF, consensus cumulative distribution function

CpG, cytosine preceding a guanosine

GBM, glioblastoma multiforme

Knn, k‐nearest neighbors

LGG, lower grade glioma

SD, standard deviation

TCGA, The Cancer Genome Atlas

WGCNA, weighted gene co-expression network analysis

**Ethics Statement**

The protocol of this article was approved by the Institutional Review Board of Hefei Institutes of Physical Science, CAS.

**Author Contributions**

XRC and ZYF: conceived and designed the experiments. CGZ, and ZYZ: collected the data. XRC, and CGZ: performed the analysis. XRC, HZW and ZYF: participated in the discussion of the algorithm. XRC and CGZ: prepared and edited the manuscript. All authors have read and approved the final manuscript.

**Funding**

This research was supported by the National Natural Science Foundation of China (81872066, [31571433](#gs1#gs1) and 81773131), the innovative program of Development Foundation of Hefei Center for Physical Science and Technology (2018CXFX004 and 2017FXCX008), and Youth Innovation Promotion Association of Chinese Academy of Sciences (2018487).

**Competing interest Statement**

The authors declare that the research was conducted in the absence of any commercial or financial relationships that could be construed as a potential conflict of interest.

**Acknowledgements**

Not applicable.

**References**

Aldape, K.D., Okcu, M.F., Bondy, M.L., and Wrensch, M. (2003). Molecular epidemiology of glioblastoma. *Cancer J* 9**,** 99-106.

Aquilanti, E., Miller, J., Santagata, S., Cahill, D.P., and Brastianos, P.K. (2018). Updates in prognostic markers for gliomas. *Neuro Oncol* 20**,** vii17-vii26.

Arber, W., and Linn, S. (1969). DNA modification and restriction. *Annu Rev Biochem* 38**,** 467-500.

Ceccarelli, M., Barthel, F.P., Malta, T.M., Sabedot, T.S., Salama, S.R., Murray, B.A., Morozova, O., Newton, Y., Radenbaugh, A., Pagnotta, S.M., Anjum, S., Wang, J., Manyam, G., Zoppoli, P., Ling, S., Rao, A.A., Grifford, M., Cherniack, A.D., Zhang, H., Poisson, L., Carlotti, C.G., Jr., Tirapelli, D.P., Rao, A., Mikkelsen, T., Lau, C.C., Yung, W.K., Rabadan, R., Huse, J., Brat, D.J., Lehman, N.L., Barnholtz-Sloan, J.S., Zheng, S., Hess, K., Rao, G., Meyerson, M., Beroukhim, R., Cooper, L., Akbani, R., Wrensch, M., Haussler, D., Aldape, K.D., Laird, P.W., Gutmann, D.H., Noushmehr, H., Iavarone, A., and Verhaak, R.G. (2016). Molecular Profiling Reveals Biologically Discrete Subsets and Pathways of Progression in Diffuse Glioma. *Cell* 164**,** 550-563.

Charlet, J., Tomari, A., Dallosso, A.R., Szemes, M., Kaselova, M., Curry, T.J., Almutairi, B., Etchevers, H.C., Mcconville, C., Malik, K.T., and Brown, K.W. (2017). Genome-wide DNA methylation analysis identifies MEGF10 as a novel epigenetically repressed candidate tumor suppressor gene in neuroblastoma. *Mol Carcinog* 56**,** 1290-1301.

Chen, E.Y., Tan, C.M., Kou, Y., Duan, Q., Wang, Z., Meirelles, G.V., Clark, N.R., and Ma'ayan, A. (2013). Enrichr: interactive and collaborative HTML5 gene list enrichment analysis tool. *BMC Bioinformatics* 14**,** 128.

Chen, X., Hao, A., Li, X., Du, Z., Li, H., Wang, H., Yang, H., and Fang, Z. (2016). Melatonin inhibits tumorigenicity of glioblastoma stem-like cells via the AKT-EZH2-STAT3 signaling axis. *J Pineal Res* 61**,** 208-217.

Crispatzu, G., Kulkarni, P., Toliat, M.R., Nurnberg, P., Herling, M., Herling, C.D., and Frommolt, P. (2017). Semi-automated cancer genome analysis using high-performance computing. *Hum Mutat* 38**,** 1325-1335.

Dawson, M.A., and Kouzarides, T. (2012). Cancer epigenetics: from mechanism to therapy. *Cell* 150**,** 12-27.

De Souza, C.F., Sabedot, T.S., Malta, T.M., Stetson, L., Morozova, O., Sokolov, A., Laird, P.W., Wiznerowicz, M., Iavarone, A., Snyder, J., Decarvalho, A., Sanborn, Z., Mcdonald, K.L., Friedman, W.A., Tirapelli, D., Poisson, L., Mikkelsen, T., Carlotti, C.G., Jr., Kalkanis, S., Zenklusen, J., Salama, S.R., Barnholtz-Sloan, J.S., and Noushmehr, H. (2018). A Distinct DNA Methylation Shift in a Subset of Glioma CpG Island Methylator Phenotypes during Tumor Recurrence. *Cell Rep* 23**,** 637-651.

Dor, Y., and Cedar, H. (2018). Principles of DNA methylation and their implications for biology and medicine. *Lancet* 392**,** 777-786.

El-Osta, A. (2004). The rise and fall of genomic methylation in cancer. *Leukemia* 18**,** 233-237.

Fanelli, M., Caprodossi, S., Ricci-Vitiani, L., Porcellini, A., Tomassoni-Ardori, F., Amatori, S., Andreoni, F., Magnani, M., De Maria, R., Santoni, A., Minucci, S., and Pelicci, P.G. (2008). Loss of pericentromeric DNA methylation pattern in human glioblastoma is associated with altered DNA methyltransferases expression and involves the stem cell compartment. *Oncogene* 27**,** 358-365.

Ghosh, A., and Barman, S. (2016). Application of Euclidean distance measurement and principal component analysis for gene identification. *Gene* 583**,** 112-120.

Gustafsson, J.R., Katsioudi, G., Degn, M., Ejlerskov, P., Issazadeh-Navikas, S., and Kornum, B.R. (2018). DNMT1 regulates expression of MHC class I in post-mitotic neurons. *Mol Brain* 11**,** 36.

Hao, X., Luo, H., Krawczyk, M., Wei, W., Wang, W., Wang, J., Flagg, K., Hou, J., Zhang, H., Yi, S., Jafari, M., Lin, D., Chung, C., Caughey, B.A., Li, G., Dhar, D., Shi, W., Zheng, L., Hou, R., Zhu, J., Zhao, L., Fu, X., Zhang, E., Zhang, C., Zhu, J.K., Karin, M., Xu, R.H., and Zhang, K. (2017). DNA methylation markers for diagnosis and prognosis of common cancers. *Proc Natl Acad Sci U S A* 114**,** 7414-7419.

Hill, V.K., Shinawi, T., Ricketts, C.J., Krex, D., Schackert, G., Bauer, J., Wei, W., Cruickshank, G., Maher, E.R., and Latif, F. (2014). Stability of the CpG island methylator phenotype during glioma progression and identification of methylated loci in secondary glioblastomas. *BMC Cancer* 14**,** 506.

Issa, J.P. (2007). DNA methylation as a therapeutic target in cancer. *Clin Cancer Res* 13**,** 1634-1637.

Jain, K.K. (2018). A Critical Overview of Targeted Therapies for Glioblastoma. *Front Oncol* 8**,** 419.

Johannessen, L.E., Brandal, P., Myklebust, T.A., Heim, S., Micci, F., and Panagopoulos, I. (2018). MGMT Gene Promoter Methylation Status - Assessment of Two Pyrosequencing Kits and Three Methylation-specific PCR Methods for their Predictive Capacity in Glioblastomas. *Cancer Genomics Proteomics* 15**,** 437-446.

Kanwal, R., Gupta, K., and Gupta, S. (2015). Cancer epigenetics: an introduction. *Methods Mol Biol* 1238**,** 3-25.

Klutstein, M., Nejman, D., Greenfield, R., and Cedar, H. (2016). DNA Methylation in Cancer and Aging. *Cancer Res* 76**,** 3446-3450.

Koch, A., Joosten, S.C., Feng, Z., De Ruijter, T.C., Draht, M.X., Melotte, V., Smits, K.M., Veeck, J., Herman, J.G., Van Neste, L., Van Criekinge, W., De Meyer, T., and Van Engeland, M. (2018). Analysis of DNA methylation in cancer: location revisited. *Nat Rev Clin Oncol* 15**,** 459-466.

Krajewska, J., Chmielik, E., and Jarzab, B. (2017). Dynamic risk stratification in the follow-up of thyroid cancer: what is still to be discovered in 2017? *Endocr Relat Cancer* 24**,** R387-R402.

Langfelder, P., and Horvath, S. (2008). WGCNA: an R package for weighted correlation network analysis. *BMC Bioinformatics* 9**,** 559.

Liu, B., Song, J., Luan, J., Sun, X., Bai, J., Wang, H., Li, A., Zhang, L., Feng, X., and Du, Z. (2016). Promoter methylation status of tumor suppressor genes and inhibition of expression of DNA methyltransferase 1 in non-small cell lung cancer. *Exp Biol Med (Maywood)* 241**,** 1531-1539.

Masci, P.G. (2017). Negative risk markers for improving prediction of heart failure: Risk stratification implementation or simply the other side of existing risk scores? *Int J Cardiol* 249**,** 328-329.

Perez, R.F., Tejedor, J.R., Bayon, G.F., Fernandez, A.F., and Fraga, M.F. (2018). Distinct chromatin signatures of DNA hypomethylation in aging and cancer. *Aging Cell* 17**,** e12744.

Verhaak, R.G., Hoadley, K.A., Purdom, E., Wang, V., Qi, Y., Wilkerson, M.D., Miller, C.R., Ding, L., Golub, T., Mesirov, J.P., Alexe, G., Lawrence, M., O'kelly, M., Tamayo, P., Weir, B.A., Gabriel, S., Winckler, W., Gupta, S., Jakkula, L., Feiler, H.S., Hodgson, J.G., James, C.D., Sarkaria, J.N., Brennan, C., Kahn, A., Spellman, P.T., Wilson, R.K., Speed, T.P., Gray, J.W., Meyerson, M., Getz, G., Perou, C.M., and Hayes, D.N. (2010). Integrated genomic analysis identifies clinically relevant subtypes of glioblastoma characterized by abnormalities in PDGFRA, IDH1, EGFR, and NF1. *Cancer Cell* 17**,** 98-110.

Weinstein, J.N., Collisson, E.A., Mills, G.B., Shaw, K.R., Ozenberger, B.A., Ellrott, K., Shmulevich, I., Sander, C., and Stuart, J.M. (2013). The Cancer Genome Atlas Pan-Cancer analysis project. *Nat Genet* 45**,** 1113-1120.

Wilkerson, M.D., and Hayes, D.N. (2010). ConsensusClusterPlus: a class discovery tool with confidence assessments and item tracking. *Bioinformatics* 26**,** 1572-1573.

Witt, H., Gramatzki, D., Hentschel, B., Pajtler, K.W., Felsberg, J., Schackert, G., Loffler, M., Capper, D., Sahm, F., Sill, M., Von Deimling, A., Kool, M., Herrlinger, U., Westphal, M., Pietsch, T., Reifenberger, G., Pfister, S.M., Tonn, J.C., and Weller, M. (2018). DNA methylation-based classification of ependymomas in adulthood: implications for diagnosis and treatment. *Neuro Oncol* 20**,** 1616-1624.

Wu, X., Rauch, T.A., Zhong, X., Bennett, W.P., Latif, F., Krex, D., and Pfeifer, G.P. (2010). CpG island hypermethylation in human astrocytomas. *Cancer Res* 70**,** 2718-2727.

Yang, H., Wu, J., Zhang, J., Yang, Z., Jin, W., Li, Y., Jin, L., Yin, L., Liu, H., and Wang, Z. (2019). Integrated bioinformatics analysis of key genes involved in progress of colon cancer. *Mol Genet Genomic Med***,** e588.

Yarus, M. (1969). Recognition of nucleotide sequences. *Annu Rev Biochem* 38**,** 841-880.

Yu, Z.Q., Zhang, B.L., Ren, Q.X., Wang, J.C., Yu, R.T., Qu, D.W., Liu, Z.H., Xiong, Y., and Gao, D.S. (2013). Changes in transcriptional factor binding capacity resulting from promoter region methylation induce aberrantly high GDNF expression in human glioma. *Mol Neurobiol* 48**,** 571-580.

Zang, L., Kondengaden, S.M., Che, F., Wang, L., and Heng, X. (2018). Potential Epigenetic-Based Therapeutic Targets for Glioma. *Front Mol Neurosci* 11**,** 408.

**Figure Legends**

### Figure 1. Flowchart Describing the Schematic Overview of the Study Design.

### Figure 2. Consensus Matrix for DNA Methylation Classification with the Corresponding Heatmap. A, Delta area curve of consensus clustering, indicating the relative change in area under the cumulative distribution function (CDF) curve for each category number **k** compared with **k** -1. The horizontal axis represents the category number **k** and the vertical axis represents the relative change in area under the CDF curve. B, Color-coded heatmap corresponding to the consensus matrix for **k**=5 obtained by applying consensus clustering. The color gradients were from 0 to 1, representing the degree of consensus, with white corresponding to 0 and dark blue to 1. C, Heatmap corresponding to the dendrogram in (B), which was generated using the pheatmap function with DNA methylation classification, tumor stage, age, and prognostic status as the annotations.

### Figure 3. Prognosis, Grade, Age Distribution, and Survival of Each Sample in the Molecular Subtypes. A, Survival curves of DNA methylation subtypes in the training set. The horizontal axis represents the survival time (days), and the vertical axis represents the probability of survival. The numbers in parentheses in the legend represent the number of samples in each cluster. The log-rank test was used to assess the statistical significance of the differences. B, Age distributions of nine DNA methylation clusters in the training set. The horizontal axis represents the DNA methylation clustering. C, Grade distributions of nine DNA methylation clusters in the training set. The horizontal axis represents the DNA methylation clustering. D, The online network tool “Enrichr” was utilized for functional enrichment analysis of genes corresponding to the gene promoter regions annotated by the CpG sites that were significantly correlated with survival.

**Figure 4. WGCNA Analysis of CpG Sites.**  **A,** Scale-free topology index and mean connectivity were used to determine the soft threshold (â=6). **B,**Clustering dendrogram of CpG sites. The dissimilarity of CpG sites is based on topological overlap. The genes are assigned to different modules and are identified using different colors. **C,** Module trait correlation analysis showed that five modules were significantly correlated with each cluster.

**Figure 5. Clustering and Survival Results of the 11 CpG Sites in the Training and Test Set. A,** Consensus clustering of the 11 CpG sites in the training set. **B,** Survival curves of two clusters predicted from the training set using the prognosis model. The log-rank test was used to assess the statistical significance of the difference. **C,** Consensus clustering of the 11 CpG sites in the test set. **D,** Survival curves of two clusters predicted from the test set using the prognosis model. The log-rank test was used to assess the statistical significance of the difference.

**Figure 6.** **Application in Clinical Practice of the Final Prognostic Predictor on 11 Feature Genes. BA,** The clinical characteristics of the 12 glioma patients. **B,** Survival curves of two clusters predicted from 12 glioma patients using the prognosis model. The log-rank test was used to assess the statistical significance of the difference. Red line indicates low methylation group (high risk group), while blue line indicates high methylation group (low risk group), based on the final prognostic predictor. **C,** ROC curve with AUC under the final prognostic predictor. **D,** The proportion of surviving glioma cells derived from glioma patients after treatment with temozolomide with indicated concentration. **E,** The proportion of surviving glioma cells derived from glioma patients after 6 Gy of irradiation. **G,** The proportion of migrated glioma cells derived from glioma patients.

**Supplementary Table 1. The Number of Samples in the 5 Clusters.**

**Supplementary Table 2. Relationship between Gene Status and DNA methylation Level in Cluster1.**

**Supplementary Table 3. Relationship between Gene Status and DNA methylation Level in Cluster2.**

**Supplementary Table 4. Relationship between Gene Status and DNA methylation Level in Cluster3.**

**Supplementary Table 5. Relationship between Gene Status and DNA methylation Level in Cluster4.**

**Supplementary Table 6. Relationship between Gene Status and DNA methylation Level in Cluster5.**

**Supplementary Table 7. The Number of Genes in the 7 Modules.**

**Supplementary Table 8. The Annotation of 11 CpG Sites.**

**Supplementary Figure 1. Comparisons of Four Properties between Training Set and Test Set Samples.** The 653 samples were evenly divided into a training set (n=327) and test set (n=326), and four properties including age (**A**, **B**), follow-up period (**C**, **D**), proportion of death samples (**E**), and tumor grade (**F**) were found to be similar in the training set and test set.

**Supplementary Figure 2. Relationship Network of the Feature Methylation Sites.**

**Supplementary Figure 3. Consensus Clustering of the 11 CpG Sites of 12 Glioma Patients.**
